# The Nonverbal Communication of Positive Emotions: An Emotion Family Approach

**DOI:** 10.1177/1754073916667236

**Published:** 2017-06-15

**Authors:** Disa A. Sauter

**Affiliations:** Department of Social Psychology, University of Amsterdam, the Netherlands

**Keywords:** emotion families, nonverbal expressions, positive emotions

## Abstract

This review provides an overview of the research on nonverbal expressions of positive emotions, organised into emotion families, that is, clusters sharing common characteristics. Epistemological positive emotions (amusement, relief, awe, and interest) are found to have distinct, recognisable displays via vocal or facial cues, while the agency-approach positive emotions (elation and pride) appear to be associated with recognisable visual, but not auditory, cues. Evidence is less strong for the prosocial emotions (love, compassion, gratitude, and admiration) in any modality other than touch, and there is little support for distinct recognisable signals of the savouring positive emotions (contentment, sensory pleasure, and desire). In closing, some limitations of extant work are noted and some proposals for future research are outlined.

A central strand of contemporary research on emotions is the study of nonverbal expressions. To date, most studies of emotional communication treat positive emotion as a unitary category, that is, “joy” or “happiness.” However, in recent years researchers are increasingly examining expressions of a range of diverse positive emotional states. This article provides an overview of that work, focusing on the study of adults (for a recent developmental review, see Sauter, McDonald, Gangi, & Messinger, 2014). This review is structured using an emotion family approach, organising the research on nonverbal signals of positive emotions into epistemological, prosocial, savouring, and agency-approach positive emotions. Epistemological emotions entail a change in one’s knowledge state, prosocial emotions emphasise a focus on others’ wellbeing, savouring emotions relate to enjoying physiologically pleasurable stimuli, and agency-approach emotions involve a tendency to approach potentially rewarding stimuli. In the final section, general conclusions and limitations are noted and some proposals for future research are outlined. In order to provide an overview, Table 1 provides a summary of the nonverbal facial, vocal, and bodily cues that have been found to occur in the communication of each of the 13 positive emotions reviewed in the current article. Table 2 gives a summary of studies that have examined nonverbal communication of specific positive emotions, with information about the channel of communication examined, whether the focus was on perception, production, or both, as well as the cultures included.

**Table 1.** Nonverbal cues found in the communication of 13 positive emotions.

Target emotion	Type of signal		
	Voice	Face	Head, body, and touch
Epistemological positive emotions			
Amusement	Laughter,^a^ vocalisations with many amplitude onsets and high spectral variation^h^	Large smile with open jaw,^cdefg^ crow’s feet^defg^	Head movement,^deg^ discontinuous touching and straight head position^i^
Awe	(Visible) inhalations^e^	Open jaw with raised inner eyebrows and widened eyes^eg^	
Interest	Fast speech rate and large vocal frequency range^b^	Parted lips,^jk^ eyelids tightened,^j^ closed^k^ or widened,^k^ raised chin,^j^ lips pressed with raised and contracted eyebrows,^g^ smile^j^	Forward leans,^gi^ head movement^gk^ facing straight ahead^i^
Relief	Sighs,^l^ vocalisations with high mean pitch and a high spectral centre of gravity and large spectral variation^h^	Smile with eyelids tightened and mouth opening^m^	Head movement up,^m^ hands in pocket^i^
Prosocial positive emotions			
Love	Low voice intensity and low pitch level,^qr^ slow speech rate^q^	Smiles with crow’s feet^gn^	Forward leans,^gn^ head nods and head movements up,^n^ affiliative hand gestures,^gop^ open posture^g^
Compassion		Oblique eyebrows,^ds^ fixed gaze^d^	Head movement forward,^ds^ forward leans,^s^ patting and stroking^p^
Gratitude		None^g^	Handshake^op^
Admiration	Conventionalised exclamations^l^		
Savouring positive emotions			
Contentment	Vocalisations of long duration and low spectral centre of gravity and high spectral variation^h^	Low-intensity smiles with crow’s feet,^g^ compressed or pressed lips^g^	Small nod^g^
Sensory pleasure	Vocalisations of long duration and low spectral centre of gravity and high spectral variation^h^	Smiles with crow’s feet and closed eyes,^jtuv^ mouth opening,^jv^ brief eyebrow raises^j^ or lowering^v^	
Sexual desire		Lip licks and bites and tongue protrusion^n^	Touching one’s lips^n^
Agency-approach positive emotions			
Elation	Conventionalised exclamations,^l^ fast speech rate and high fundamental frequency and high mean energy^b^	Smiles^jtuw^ with open mouth,^jw^ widened eyes,^tu^ raised eyebrows and chin^j^	Fast, expansive movements with stretched out arms and tilted head,^x^ repetitive vertical arm and knee movements^i^
Pride		Small smile,^y^ crow’s feet, parted lips and raised chin^j^	Expanded posture with head tilted slightly back and arms out,^yzaa^ symmetrical vertical arm movements^i^

*Note.* All sources can be found in the reference list. ^a^Ruch (1995); ^b^Banse and Scherer (1996); ^c^Ambadar et al. (2009); ^d^Haidt and Keltner (1999); ^e^Shiota et al. (2003); ^f^Hess et al. (2002); ^g^Campos et al. (2013); ^h^Sauter, Eisner, Calder, et al. (2010); ^i^Dael et al. (2012); ^j^Mortillaro et al. (2011); ^k^Reeve (1993); ^l^Schröder (2003); ^m^Krumhuber and Scherer (2011); ^n^Gonzaga et al. (2001); ^o^Hertenstein et al. (2009); ^p^Hertenstein et al. (2006); ^q^Juslin and Laukka (2003); ^r^Hammerschmidt and Jürgens (2007); ^s^Eisenberg et al. (1989); ^t^Ricci-Bitti et al. (1996); ^u^Wehrle et al. (2000); ^v^Fernandez-Dols et al. (2011); ^w^Fujimura and Suzuki (2010); ^x^Wallbott (1998); ^y^Tracy et al. (2014); ^z^Tracy and Matsmoto (2008); ^aa^Tracy and Robins (2008).

**Table 2.** Behavioural studies that have examined nonverbal communication of specific positive emotions.

Study	Signal	Perception/production	Culture(s)	Emotions examined
Ambadar et al. (2009)	F	Perception	USA	**Amusement**, **politeness**, embarrassment, nervousness
App et al. (2011)	F, B, T	Both	USA	**Happiness, love, pride, sympathy**, anger, disgust, embarrassment, fear, guilt, shame, sadness
Banse and Scherer (1996)	S	Both	Germany	**Elation, happiness, interest, pride**, hot anger, cold anger, panic fear, anxiety, despair, sadness, boredom, disgust, contempt, shame
Bänziger et al. (2012)	S, F+B, AV	Perception	Switzerland	**Amusement, pride, joy, relief, interest, pleasure, admiration, tenderness**, hot anger, panic, fear, despair, irritation, anxiety, sadness, disgust, contempt, surprise
Campos et al. (2013)	F+B	Production	USA	**Amusement, awe, contentment, gratitude, interest, joy, love, pride**
Cordaro et al. (2016)	V	Perception	Production: USA; perception: China, Germany, India, Japan, South Korea, New Zealand, Pakistan, Poland, Turkey, USA, Bhutan	**Awe, triumph, interest, amusement, contentment, desire, relief, compassion**, anger, disgust, fear, sadness, surprise, pain, contempt, embarrassment
Cowie and Cornelius (2003)	S	Perception*	UK	**Amusement, pleasure, happiness, excitement, confidence, interest, affection, love, contentment, relaxation**, neutral, anger, sadness, worry, boredom, disappointment, fear
Dael et al. (2012)	B	Production	Switzerland	**Elated joy, amusement, pride, pleasure, relief, interest**, rage, panic, fear, despair, irritation, worry, sadness
De Meijer (1989)	B	Perception	The Netherlands	**Joy, interest, sympathy, admiration**, grief, anger, fear, surprise, disgust, shame, contempt, antipathy
Gonzaga et al. (2001)	F+B	Production*	USA	**Love, desire, happiness**
Gonzaga et al. (2006)	F+B	Production*	USA	**Love, sexual desire**
Haidt and Keltner (1999)	F	Perception	Production: USA; perception: USA, India	**Happiness, amusement, sympathy**, anger, fear, sadness, disgust, surprise, contempt, embarrassment, shame
Hawk et al. (2009)	F, V, S	Perception	The Netherlands	**Joy, pride**, sadness, surprise, neutral, anger, contempt, disgust, fear, embarrassment
Hammerschmidt and Jürgens (2007)	S	Production	Germany	**Joyful surprise, sensual satisfaction, affection**, rage, despair, disgust
Harris and Alvarado (2005)	F	Production*	USA	**Amusement, tickle**, pain
Hejmadi et al. (2000)	B	Perception	Production: India; perception: India, USA	**Heroism, amusement, love, peace, wonder**, anger, disgust, fear, sadness, embarrassment
Hertenstein et al. (2009)	T	Both	USA	**Love, gratitude, sympathy, happiness**, anger, fear, sadness, disgust
Hertenstein et al. (2006)	T	Both	Spain, USA	**Love, envy, pride, gratitude, happiness, sympathy**, anger, disgust, fear, sadness, surprise, embarrassment
Hess et al. (2002)	F	Perception	Canada	**Appeasement, amusement**, dominance
Krumhuber and Scherer (2011)	F	Production	Switzerland	**Joy, relief**, anger, fear, sadness
Laukka et al. (2013)	V	Perception	Production: India, Kenya, Singapore, USA; perception: Sweden	**Affection, happiness, interest, desire, serenity, pride, relief, positive surprise**
Mortillaro et al. (2011)	F	Production	Switzerland	**Pride, interest, pleasure, joy**
Ricci-Bitti et al. (1996)	F	Both	Italy	**Sensory pleasure, joy, elation**, formal unfelt
Sauter and Scott (2007)	V	Perception	Production: UK; perception: Sweden, UK	**Triumph, amusement, pleasure, relief, contentment**
Sauter, Eisner, Calder, et al. (2010)	V	Both	UK	**Triumph, amusement, pleasure, relief, contentment**, anger, fear, disgust, sadness, surprise
Sauter, Eisner, Ekman, et al. (2010)	V	Perception	Production: UK, Namibia; perception: UK, Namibia	**Triumph, pleasure, amusement, relief**, anger, fear, disgust, sadness, surprise
Schröder (2003)	V	Both	Germany	**Admiration, elation, relief**, threat, disgust, boredom, startle, worry, contempt, rage
Shiota et al. (2003)	F	Production	USA	**Awe, amusement, pride**
Simon-Thomas et al. (2009)	V	Perception	USA	**Amusement, awe, interest, relief, compassion, gratitude, love, contentment, desire, sensory pleasure, enthusiasm, pride, triumph**
Szameitat et al. (2009)	V	Perception	UK	**Tickle, joy**, taunt, schadenfreude
Tracy and Matsumoto (2008)	F+B	Production*	36 nations	**Pride, joy**, shame, sadness, fear, anger, disgust
Tracy and Robins (2008)	F+B	Perception	Italy, Burkina Faso, USA	**Pride, happiness**, anger, disgust, fear, sadness, shame, surprise
Wallbott (1998)	B	Production	Germany	**Elated joy, pride, happiness**, sadness, despair, fear, terror, cold anger, hot anger, disgust, contempt, shame, guilt, boredom
Wehrle et al. (2000)	F	Perception	Switzerland	**Happiness, elation, pleasure**, cold anger, hot anger, sadness, desperation, anxiety, fear

*Note.* Only studies that have examined multiple positive emotional states within a single study are included. Production denotes studies that include measures of the physical cues of the expressions, regardless of the method of elicitation. Stars denote studies that employed spontaneously produced expressions. Positive emotions are in bold. Where not specified in the original article, culture is inferred from authors’ affiliations. Abbreviations: S = speech intonation; V = vocalisations; F = facial expressions; B = bodily cues; T = touch; AV = audiovisual.

## Mapping Emotions to Expressions

One feature of the investigation of nonverbal expressions of emotions is the use of objective cues like acoustic information and analyses of facial and bodily muscle movements. In mapping emotional states onto physical cues, researchers attempt to establish links between emotion and behaviour without relying on subjective self-report as a primary measure. Emotions are typically either inferred from antecedent events or known in advance when expressions are posed. A complementary approach to measuring objective cues employs perceptual measures such as ratings or classification tasks, to establish how perceivers judge others’ emotional expressions. The majority of studies to date have examined the perception, rather than production, of emotional expressions. The logic is that perceivers can only consistently map an emotional state to an observed nonverbal expression if there is a link between an emotional state and that expression in the expresser. For example, if observers consistently infer that a person who is laughing is amused, that is taken to demonstrate a link between laughter and amusement, which is assumed to exist not only in the observer but also in the expresser.

Though a wealth of empirical evidence points to consistent mappings between nonverbal expressions and subjective emotional states for a limited set of emotions (see Lench, Flores, & Bench, 2011, for a meta-analysis) considerable variability has also been noted (Scarantino, 2015). Emotional expressions do not always occur when an emotional state is experienced, and conversely, some configurations of nonverbal behaviours occur despite an individual not experiencing the emotional state that the expression supposedly maps onto. Recent accounts claiming links between emotional states and nonverbal signals have emphasised the probabilistic nature of these associations (see Levenson, 2011; Roseman, 2011). Only when unlearned triggers occur would the link to behaviour (including nonverbal expressions) be rigid (Ekman, 2007). For example, experiencing a sudden loss of physical support would be linked to fear behaviours, and tasting a strong bitter flavour would elicit disgust behaviours. For positive emotions, unlearned triggers may include gentle touch and sweet tastes. The probability of a fixed association between subjective state and nonverbal behaviour is thought to depend on the prototypicality of the antecedent event, and relatedly, on the intensity of the emotional experience (Scarantino, 2015). Importantly for the current discussion, however, a claim of an association between a nonverbal behaviour and a subjective emotional state does not depend on a perfect one-to-one mapping between the two.

## Emotion Families

How best to conceptualise the structure of human emotions is an issue on which views range widely. Nevertheless, most theorists would agree that each emotional state is not equally similar to all other emotional states. One way to operationalise these similarities is the notion of “emotion families” proposed by Ekman (1992), that is, groups of emotional states sharing common characteristics. Such clustering could be done on the basis of features such as antecedent events, nonverbal expressions, or patterns of appraisals or action tendencies.

Most research to date has presumably examined only a single positive emotion (“happiness”) because all positive emotions have been considered part of a single emotion family, as positive emotions are all characterised by positive valence. Some accounts have posited shared mechanisms of positive emotions, such as facilitating approach (Davidson & Irwin, 1999) or increasing one’s repertoire of thoughts and actions (Fredrickson, 1998). An alternative possibility is that positive emotion space is comprised of multiple emotion families that share additional characteristics. An analysis of the English emotion lexicon has lent support to this kind of structure: words cluster together around multiple positive emotional concepts (Shaver, Schwartz, Kirson, & O’Connor, 1987), and there is some preliminary evidence suggesting that nonverbal behaviours may also cluster into conceptually meaningful positive emotion families (App, McIntosh, Reed, & Hertenstein, 2011; Simon-Thomas, Keltner, Sauter, Sinicropi-Yao, & Abramson, 2009). Building on this work, the current review is organised in sections of possible families of positive emotions (Simon-Thomas et al., 2009), discussing nonverbal expressions of epistemological, prosocial, savouring, and agency-approach positive emotions in turn. However, given the scarcity of proposed classifications of positive emotions, this division is necessarily preliminary. This is true both in terms of whether specific emotion categories are best classified into one or the other superordinate category, and in terms of whether this structure is useful for establishing commonalities across subsets of positive emotions. In addition to providing an overview of extant research, one aim of the current review is to evaluate whether this proposed classification fits the available evidence on nonverbal communication of emotions.

## Epistemological Positive Emotions

Some positive emotional states involve a change in the individual’s understanding of, or knowledge about, the world. These emotions can be considered epistemological positive emotions. They can, for example, involve the seeking out of new information (i.e., interest), or the realisation that an expected negative event will not occur (i.e., relief). The new information need not in itself be positive, but the change in knowledge results in a positive emotional state. The epistemological positive emotions include interest, relief, amusement, and awe.

### Amusement

Amusement, the feeling of finding something funny, is a positive subjective state that can result from a resolution of incongruity (Carroll, 2013). In recent years, the nonverbal behaviour of amusement has been the focus of a considerable body of research, both in studies examining auditory and visual communication. This research has primarily examined laughter, a vocal and facial suite of behaviours associated with amusement (for reviews on laughter see Owren & Amoss, 2014; Ruch & Ekman, 2001). Experimental work has established that amusement induction (e.g., funny movies) reliably induces laughter (e.g., Ruch, 1995), though most research has focused on whether observers infer amusement from others’ laughter. In a recent study using a multimodal corpus of emotional expressions, amusement was found to be the best recognised of all 12 emotions studied across modalities (Bänziger, Mortillaro, & Scherer, 2012). The demonstration by Bänziger and colleagues that laughter can be recognised from visual cues alone extends earlier findings that have described the facial movements associated with amusement without directly linking production and perception. Several studies have noted that amusement is linked to Duchenne smiles, that is, smiles co-occurring with raised cheeks (Campos, Shiota, Keltner, Gonzaga, & Goetz, 2013; Hess, Beaupré, & Cheung, 2002; Shiota, Campos, & Keltner, 2003). In particular, spontaneous amusement has been linked to intense smiles with open jaws, and perceivers also judge smiles with those characteristics as expressing amusement (Ambadar, Cohn, & Reed, 2009). Notably, this configuration of cues has been found to communicate amusement across several cultures (Haidt & Keltner, 1999).

A small body of research has examined full-body cues associated with emotions, with recent findings suggesting that bodily cues can under some conditions convey affective information more clearly than facial expressions (Aviezer, Trope, & Todorov, 2012). Data is lacking for most positive emotions, but a recent study examined the bodily configuration associated with amusement as well as a few additional positive emotions (Dael, Mortillaro, & Scherer, 2012). Employing discriminant analyses of body movements, configurations of movements expressing amusement were accurately classified, and evidence for a prototypical response pattern was established. Specifically, discontinuous touching and a straight head position was found to be characteristic of amusement, and the authors suggested that this likely reflects a laughter response pattern involving the entire body.

Laughter has also been found to communicate amusement via auditory perception alone (see Owren & Amoss, 2014, for a review) via both nonverbal vocalisations and speech inflection (e.g., Cowie & Cornelius, 2003; Sauter, Eisner, Calder, & Scott, 2010; Simon-Thomas et al., 2009). Notably, this pattern is consistent across cultures (Cordaro, Keltner, Tshering, Wangchuk, & Flynn, 2016; Laukka et al., 2013; Sauter, Eisner, Ekman, & Scott, 2010; Sauter & Scott, 2007). In sum, amusement can be clearly communicated using either visual or auditory cues of laughter across cultures. Laughter has been linked to emotional states other than amusement, including schadenfreude (Szameitat et al., 2009), and is part of the response to the tactile stimulation of being tickled, which may or may not be accompanied by an emotional state (see Harris & Alvarado, 2005). Nevertheless, the link between the emotional state of amusement and nonverbal signals of laughter appears to be robust across modalities and cultural groups.

### Awe

There is growing interest from emotion researchers in awe, the feeling of being in the presence of something greater than oneself (see Stellar et al., 2017; Valdesolo et al., 2017). Awe, which is often elicited by views of nature, has been suggested to involve a need for cognitive accommodation, that is, the adjustment of one’s ideas of what is possible in the world (Keltner & Haidt, 2002). However, only a few studies have investigated the nonverbal communication of awe, though there has been considerable consistency across those studies. Examinations of facial expressions of awe have shown that smiling rarely occurs, but rather, awe is associated with head movements forward and up, widened eyes, an open mouth with a slightly dropped jaw, and raised inner eyebrows (Campos et al., 2013; Shiota et al., 2003). These facial changes may in part facilitate the hypothesised function of awe, that is, the enhanced processing of information to aid the cognitive accommodation sought during experiences of awe. However, no study to date has examined the recognition of facial signals of awe, though one study has found high levels of recognition across two cultural groups for Indian dance segments expressing the closely related state wonder (Hejmadi, Davidson, & Rozin, 2000).

Participants posing awe expressions also frequently produced visible inhalations (Shiota et al., 2003), and awe has also been associated with voiced exhalations (Simon-Thomas et al., 2009). Such prototypical vocal awe displays are well recognised by naive listeners (Simon-Thomas et al., 2009). This suggests that awe is reliably communicated via auditory signals, in addition to having a consistent configuration of facial cues. It is also worth noting that awe has been linked to goosebumps, which have been posited to serve a signalling function for profound positive experiences (Maruskin, Thrash, & Elliot, 2012); that positive emotion can be inferred from perceiving goosebumps may be an interesting hypothesis for further study.

### Interest

Interest, the feeling of wanting to learn more about something, functions to motivate exploration and has been proposed to be a primary affect (Tomkins, 1995). However, results on the nonverbal communication of interest are conflicting. For example, interest being associated with an open mouth (Mortillaro, Mehu, & Scherer, 2011) is contradicted by results linking interest to lip presses (Campos et al., 2013). Furthermore, in Bänziger et al.’s (2012) multimodal study, interest was overall the worst recognised positive emotion out of the six included in the study, with speech intonation even less well recognised than facial displays. This contrasts with earlier findings that have found high levels of recognition for interested speech prosody, characterised by fast rate of speech and a great vocal frequency range (Banse & Scherer, 1996). There is more consistency in the results of studies of nonverbal vocalisations, with exclamations of interest recognisable (Simon-Thomas et al., 2009) even across cultural boundaries (Laukka et al., 2013).

In terms of full body movements, interest is characterised by facing straight ahead and leaning forward, likely linked to the motivation to approach associated with interest (Dael et al., 2012; see also de Meijer, 1989). However, this bodily configuration is not unique to interest and expressions are sometimes misclassified as pride.

It has been suggested that awe and interest may be best explained as variations of a single emotion, as both involve some degree of cognitive accommodation, though interest is thought to be less intense (Campos et al., 2013). However, the only recognition study to date to include both awe and interest found low rates of confusion between the two for nonverbal vocalisations (Simon-Thomas et al., 2009). Could it be that there is more overlap between the facial than vocal configurations of awe and interest? Facial expressions of interest, like those of awe, do not typically involve smiling, but rather an open mouth (Mortillaro et al., 2011; Reeve, 1993). However, the eye configuration often seen in awe with wide open eyes stands in contrast to the mild squinting or eye closure associated with interest (Mortillaro et al., 2011; Reeve, 1993). This suggests some differentiation, but at present, it is not known whether perceivers can differentiate between facial expressions of awe and interest. In sum, there is evidence for recognisable nonverbal vocalisations of interest, but evidence on facial expressions is less clear-cut, in particular with regard to the relationship between awe and interest configurations.

### Relief

Relief is a positive emotional experience that occurs when an unpleasant emotional experience ceases. If the unpleasant experience is ongoing, relief is elicited when it ends, or upon learning that it will end sooner than expected. If the negative experience is anticipated to occur in the future, relief results from finding out that the negative experience will not occur. Relief can also be the result of a negative experience being less bad than had been expected. Multiple studies of nonverbal vocalisations have established that relief can be very reliably inferred from sighs. For example, Schröder (2003) found that relief was recognised at near-ceiling levels by listeners. This finding has since been replicated (Sauter, Eisner, Calder, et al., 2010; Simon-Thomas et al., 2009) and extended to show that relief elicits sighs across cultures (Laukka et al., 2013; Sauter, Eisner, Ekman, et al., 2010).

Relief can also be recognised from visual cues alone (Bänziger et al., 2012), though no work has yet tested whether visual cues of relief are consistent across cultures. Facially, prototypical relief expressions are characterised by a low-intensity smile, preceded by mouth opening, eye closure, and the head moving up (Krumhuber & Scherer, 2011). In terms of global body movements, only one study to date has included relief (Dael et al., 2012). A statistical classifier could accurately classify relief expressions and differentiate them from other (positive) emotions, but the only distinguishing feature of the relief expressions was that of retiring one’s hands in one’s pockets.

### Summary: Epistemological Positive Emotions

To summarise, there is clear support that all of the epistemological positive emotions examined to date (amusement, relief, awe, and interest) have distinct, recognisable displays via vocal or facial cues. These, together with pride (see section below) arguably constitute the strongest candidates for positive emotions associated with specific, identifiable nonverbal expressions.

## Prosocial Positive Emotions

Positive emotions can serve the function of orienting people towards the welfare of others and to foster profound social relationships. It has been argued that increased concern for others is a central aspect of many positive emotions, though it is particularly pronounced for certain emotions (Keltner, 2009). The prosocial positive emotions include love, compassion (sometimes called sympathy), gratitude, and admiration. These emotions stand in contrast to positive emotions that enhance the individual experiencing the emotion, such as pride.

### Love

A consensus definition of love is lacking in the literature, and both whether it is an emotion and whether it is positive have been questioned (Lamy, 2016). Nevertheless, love is typically conceptualised as a positive emotion that stimulates com-mitment to intimate relationships (Campos et al., 2013). Nonverbal signals of love may reward prosocial behaviour and signal prosocial intent (Hertenstein, Keltner, App, Bulleit, & Jaskolka, 2006).

Several studies have established an association between bodily movements and love. One study examined couples in romantic relationships as they interacted with each other in a series of semistructured discussions. Self-reported feelings of love correlated with head nods, Duchenne smiles, and forward leans (Gonzaga, Keltner, Londahl, & Smith, 2001). Consistent with these results, a study examining posed expressions found that love was associated with Duchenne smiles, mutual gaze, affiliative hand gestures, open posture, and forward leans (Campos et al., 2013). It is notable that these studies have highlighted movements beyond the face, such as postural shifts and hand gestures. However, love may in fact be preferentially communicated via a completely different type of signal, namely touch (App et al., 2011). App and colleagues found that, when given an unrestricted choice of expression modality between touch, face, or body, touch was preferred to bodily or facial cues for expressing love, and signals of love were identified more accurately from observed touch than from facial or bodily movement. This corroborates studies showing that love can be reliably communicated from touch, typically using hugging or stroking movements (Hertenstein, Holmes, McCullough, & Keltner, 2009), in both North American and Spanish participants (Hertenstein et al., 2006).

In contrast, the evidence that love can be communicated via vocal signals is less strong. A meta-analysis of vocal communication of emotion grouped positive emotions into “happiness” and “love-tenderness” (Juslin & Laukka, 2003). Speech inflected with love was characterised by slow speech rate, low voice intensity, low pitch level, and little pitch variability, but had the lowest decoding levels of all the emotions examined. This aligns with findings from the communication of emotions via nonverbal vocalisations, where only modest recognition rates have been found for vocalisations of love (affection), both within and across cultures (Laukka et al., 2013). The authors concluded that love may lack a distinct type of vocalisation. Furthermore, affection vocalisations were commonly confused with interest, an epistemological rather than prosocial emotion. Frequent misclassifications of speech intonation pattern of tenderness have also been found in a study with a statistical classifier model using acoustic information (Hammerschmidt & Jürgens, 2007). Speech expressing tenderness was specifically misclassified as expressing sensual satisfaction, which could be considered a savouring positive emotion. Thus, there is evidence that love is communicated via touch and full-body movement, but it does not appear to be associated with unique, clearly recognisable vocal cues.

### Compassion

Compassion, sometimes referred to as sympathy, is a desire to help in response to perceiving another’s suffering (see Stellar et al., 2017). It is differentiated from empathy, which is the vicarious experiencing of another’s suffering, because feeling compassion does not necessarily involve suffering and theorists have conceptualised it as a positive emotional experience (Goetz, Keltner, & Simon-Thomas, 2010). Several studies have shown that compassion is marked by some facial features of sadness, combined with approach behaviours such as forward leans (Eisenberg et al., 1989; see also de Meijer, 1989). This differs from facial displays of love because of the absence of smiling, and differs from sadness due to the signals of approach in compassion. However, visual expressions of compassion are poorly recognised and often mistaken for sadness (e.g., Haidt & Keltner, 1999).

Consistent with the pattern of results for visual displays, the study of vocal expressions has found low levels of accuracy for recognition of nonverbal vocalisations of compassion (Simon-Thomas et al., 2009). An analysis of classification errors revealed that love and gratitude vocalisations were often identified as expressions of compassion, suggesting that vocalisations of all of those emotions may be used to communicate a general prosocial state of affiliation.

The strongest evidence for signals of compassion comes from the study of touch, with patting and stroking movements recognised as expressions of compassion across two cultural groups (Hertenstein et al., 2006). This is supported by a direct comparison across channels of communication, where participants favoured touch over face and body for expressing compassion (App et al., 2011). Naive participants were also more accurate in identifying compassion from observed touch as compared to facial and bodily displays, and in line with findings from studies of recognition from vocal cues, errors classifying touch tended to occur between compassion and love. In sum, evidence for a distinct, recognisable display of compassion from visual or auditory cues is weak, but rather, compassion appears to be preferentially signalled via touch.

### Gratitude

Gratitude is what we feel when someone lends us a helping hand. It has been conceptualised as a positive emotional state caused by appreciating benefits perceived to be intentionally bestowed upon oneself, and it is thought to be important for promoting social relationships (Algoe & Haidt, 2009; see also Armenta et al., 2017; Stellar et al., 2017). Though gratitude is marked by behavioural tendencies, such as reciprocating (Algoe & Haidt, 2009), little research has examined nonverbal expressions of this prosocial emotion and results to date are weak.

Gratitude has been found to lack a reliable visual expressive display (Campos et al., 2013) and to be poorly recognised from nonverbal vocalisations (Simon-Thomas et al., 2009). In contrast, touch has been shown to communicate gratitude (Hertenstein et al., 2009; Hertenstein et al., 2006), but the most frequent way to express gratitude was a handshake, suggesting a conventionalised signal that may not generalise beyond particular cultural groups.

### Admiration

The experience of admiration is triggered by the perception of another’s extraordinary achievement (Algoe & Haidt, 2009). We may feel admiration when learning of someone having a brilliant insight, or when experiencing an exceptional artistic accomplishment, such as a virtuoso musical performance. Said achievement should be outside of the moral domain, as witnessing moral virtue triggers elevation (Algoe & Haidt, 2009), which has not yet been studied in the context of nonverbal communication. Admiration is a positive emotion that has received little attention from researchers of nonverbal communication, and the available evidence holds only limited promise for the notion that admiration may be signalled via nonverbal expressions.

In a study of nonverbal vocalisations, Schröder (2003) found that recognition levels were nearly at ceiling for expressions of admiration. However, this was also the case when only segmental information was provided (i.e., transcriptions such as “wow”), suggesting that these exclamations may be conventionalised emblems, rather than fully nonverbal vocalisations like sighs and screams.

In a more recent study by Bänziger et al. (2012), moderate levels of recognition were found from both visual and auditory signals of admiration when presented alone, but there was a marked advantage for audiovisual presentation. This may suggest that there is relatively little redundancy in expressions of admiration, such that visual and auditory cues complement each other. Notably, expressions of admiration were frequently mistaken for surprise, which likely reflects the conceptual overlap between these two emotions, as both involve an unexpected event (Algoe & Haidt, 2009).

### Summary: Prosocial Positive Emotions

Current evidence indicates that the prosocial emotions (love, compassion, gratitude, and admiration) are not reliably communicated in any modality other than touch. This is clear support for the proposal that the most effective mode of communication of an emotion may depend in part on the emotion’s social function (App et al., 2011), with prosocial emotions playing a particularly important role in intimate social relationships.

## Savouring positive emotions

An obvious way that positive emotions are triggered is from thinking about or experiencing enjoyable stimuli such as food or sex. Such savouring positive emotions include contentment, sensory pleasure, and sexual desire. The roots of savouring emotions are likely linked to unconditioned stimuli like food and touch, that fulfil basic needs.

### Contentment

Contentment has been defined as an emotion accompanying satisfaction of one’s basic needs. Sometimes called satisfaction, it is the feeling of enjoying a quiet rest after completing a good day’s work. The central appraisal features that characterise contentment do not differentiate it well from other positive emotions (Campos et al., 2013). Conflation between contentment and other positive emotional states has also been found in the study of vocal signals. Specifically, nonverbal vocalisations of contentment were often confused with (sensual) pleasure across several studies (Sauter, Eisner, Calder, et al., 2010; Sauter & Scott, 2007). It is not clear whether visual cues of contentment are specific to that emotion; posed displays have been shown to primarily feature smiling (Campos et al., 2013). Specifically, Duchenne smiles and smiles with the lips pressed together occurred frequently in expressions of contentment, but given the ubiquity of smiling in positive emotions it remains to be established whether facial expressions of contentment can be recognised.

### Sensory Pleasure

Sensory pleasure is the enjoyment of a physical stimulus, such as food or sex. Sensory pleasure can be elicited from unconditional stimuli such as pleasant touch, but also through learned associations. For example, one can experience pleasure from tasting flavours that are at first exposure not typically judged to be pleasant (e.g., coffee, wine). Although pleasure has been included in a number of studies of nonverbal behaviour, results to date do not clearly point to a unique, recognisable signal for this emotional state.

As noted, vocal signals of pleasure are frequently confused with contentment (e.g., Sauter & Scott, 2007), and though recognition rates for pleasure are relatively high within culture, they appear to be culturally variable (Sauter, Eisner, Ekman, et al., 2010). However, given that posed expressions of pleasure are perceived as inauthentic (Bänziger et al., 2012), it remains to be tested whether genuine-sounding vocalisations of pleasure are shared across cultural boundaries.

In terms of bodily movements, pleasure has been found to be associated with a prototypical response pattern consisting of tilting the head upwards and away, accompanied by asymmetrical arm movements (Dael et al., 2012). However, no study to date has examined whether observers can infer pleasure from others’ bodily movements.

Examining facial cues of positive emotions, Ricci-Bitti, Caterina, and Garotti (1996) described the facial changes associated with pleasure as closed eyes in combination with a Duchenne smile. This was corroborated by a more recent study (Mortillaro et al., 2011), which found that facial expressions of pleasure were characterised by smiling, eye closure, and mouth opening. Complementing this work, there is some evidence suggesting that perceivers can recognise pleasure from facial cues. However, this seems to be the case primarily for pleasure expressions of high intensity (Wehrle, Kaiser, Schmidt, & Scherer, 2000) and for audiovisual displays (Bänziger et al., 2012). It is worth noting that confusion patterns for pleasure expressions are highly variable; they have been interpreted primarily as love/tenderness (Bänziger et al., 2012), interest (Mortillaro et al., 2011), and pride (Wehrle et al., 2000). The variation in emotion stimuli used in these studies renders these inconsistencies difficult to interpret, and highlight the need for consistency in studies in this area. One contributing factor, however, may be that the emotion most closely related to pleasure, at least for facial expressions, is not another positive emotion, but rather pain. Detailed analysis of facial movements during pleasure and pain have supported the notion that there is overlap between the facial configurations of these two states (Fernandez-Dols, Carrera, & Crivelli, 2011), though observers are able to distinguish these expressions at greater than chance levels in the absence of contextual cues (Hughes & Nicholson, 2008). However, the studies examining overlap in facial expressions between pleasure and pain have specifically focused on sexual pleasure; sexual enjoyment may differ from other forms of sensory pleasure, both in terms of facial configurations and on other features, such as action tendencies.

### Sexual Desire

Desire is a state that leads a person to seek out opportunities for sexual activity (Gonzaga, Turner, Keltner, Campos, & Altemus, 2006). It differs from sensory pleasure, which may also contain a sexual element, in that the contact has not yet occurred. Only a few studies have examined the nonverbal expressions of sexual desire.

Just a single study to date has examined the visual cues of desire (Gonzaga et al., 2006). It showed that self-reported feelings of desire in a semistructured interaction correlated with lip licks, bites, and puckering during interactions with a romantic, sexual partner. These sexual displays were also correlated with the partner’s self-reported sexual desire, but whether these displays are explicitly recognised as signalling desire has not yet been tested.

Evidence on vocal signals of desire is mixed. One study found that nonverbal vocalisations of desire were poorly recognised, with errors distributed across many other response alternatives, possibly due to large variability among the desire expressions (Simon-Thomas et al., 2009). In contrast, Laukka et al. (2013) found that lust was one of the best recognised vocalisation types of the nine positive emotions in their study. Recognition levels were however still moderate, because sounds were frequently conflated with relief, serenity, and positive surprise. It may be that the inconsistency between these studies can be accounted for by differences between desire and lust, but it cannot currently be concluded that desire is associated with a nonverbal signal in any modality.

### Summary: Savouring Positive Emotions

There is currently little support for distinct recognisable signals of the savouring positive emotions (contentment, sensory pleasure, and desire). The overlap between them in terms of confusion errors may be an indication that these do not constitute states with distinct signals; the extent to which other criteria do differentiate these emotional states will be a worthwhile question for future research.

## Agency-Approach Positive Emotions

Though not all positive emotions facilitate approach towards reward-related actions (e.g., Gable & Harmon-Jones, 2008), some, including elation and pride, are characterised by approach tendencies. These emotions are more individual than many of the other positive emotions, that is, they do not primarily involve a positive interaction with another person: they may not require the involvement of a social partner at all (i.e., elation) or they may involve others only for social comparison (i.e., pride).

### Elation

Elation is a highly aroused positive emotional state caused by an unexpected positive event (Mortillaro et al., 2011), such as winning the lottery. Closely related states are enthusiasm, triumph, and excitement; these are discussed together here as there is currently little theoretical differentiation made between these states. Elation is however differentiated from joy (sometimes called happiness), which typically refers to a positive emotional state in a more general sense. Elation is distinct from joy both in terms of the specificity of the target state, and in terms of levels of arousal, since elation is characterised by particularly high arousal.

In a study of emotional speech prosody comparing segments differing in arousal, most emotion pairs (e.g., panic and anxiety) were found to frequently be confused (Banse & Scherer, 1996). However, elation and joy were rarely mistaken for each other, suggesting that they may have distinct prosodic contours, although recognition levels for both were moderate.

Several studies have demonstrated that nonverbal vocalisations of enthusiasm and triumph are not well recognised, and it has been suggested that this emotional state may lack a clear vocal nonverbal signature (Schröder, 2003). Consistent with this notion, Bänziger et al. (2012) found that vocal signals of elated joy were poorly recognised, and frequently confused with amusement. Simon-Thomas et al. (2009) found that elation in the form of triumph was poorly recognised from nonverbal vocalisations, while enthusiasm was recognised with moderate levels of accuracy. Notably, enthusiasm and triumph were frequently mistaken for each other, underscoring the overlap between these two states.

Facial expressions of elation are marked by smiles accompanied by widened eye aperture (Ricci-Bitti et al., 1996) or Duchenne smile together with raised eyebrows (Mortillaro et al., 2011). In terms of recognition, judges can differentiate elated facial expressions well from related states such as pleasure and happiness, especially when viewing high-intensity, dynamic expressions (Wehrle et al., 2000; see also Fujimura & Suzuki, 2010, for a similar finding).

Several studies have examined full-body cues associated with elation. Wallbott (1998) found that elation was characterised by high movement activity and fast and expansive movements (see also de Meijer, 1989, for a similar result). Specifically, the arms were stretched out and the head was tilted up and back. A discriminant analysis yielded high levels of accuracy for elevated joy expressions. Consistent with these findings, a more recent study found high accuracy for full-body cues submitted to a discriminant analysis, though the specific movements for elated joy were repetitive vertical movement of the arms and knees (Dael et al., 2012). However, recognition data from full-body cues associated with elated joy is currently missing.

In sum, elation is linked to reliable patterns of facial and bodily movements, but not to a type of nonverbal vocalisation.

### Pride

Pride is a positive, self-conscious emotion triggered by the completion of a goal; it is thought to play a role in enhancing the individual’s social status (Tracy & Robins, 2007). We feel proud when accomplishing a challenging goal, be it academic, athletic, or personal. Pride is the positive emotion whose nonverbal communication has been studied most extensively (see Tracy, Weidman, Cheng, & Martens, 2014, for a review).

Evidence for consistent and recognisable visual displays of pride is strong. Prototypical displays are characterised by a combination of bodily and facial changes, specifically expanded posture, a head tilt back, and a small smile (Tracy et al., 2014). This expression is produced spontaneously in pride-eliciting situations and communicates pride also across cultural boundaries (Tracy & Matsumoto, 2008; Tracy & Robins, 2008). Notably, facial cues alone are insufficient for perceivers to differentiate pride from joy (App et al., 2011; Mortillaro et al., 2011; Wehrle et al., 2000) and conversely, pride is frequently misclassified as elation when bodily cues of pride are judged in the absence of facial information (Dael et al., 2012; Wallbott, 1998).

In contrast, several studies have shown that pride is not well recognised from auditory signals, with classification errors distributed across both positive and negative emotions (Bänziger et al., 2012; Simon-Thomas et al., 2009).

To summarise, pride is communicated via a combination of postural and bodily cues, but not from vocalisations or facial expressions alone.

### Summary: Agency-Approach Positive Emotions

The findings available to date suggest that agency-approach positive emotions (elation and pride) are associated with recognisable visual, but not auditory, cues. The consistency seen for the emotions in this emotion family may point to positive emotions characterised by a strong approach motivation being preferentially communicated via visual signals.

## Conclusions, Limitations, and a Look to the Future

This article has provided an overview of the literature on the nonverbal communication of positive emotions. This research provides support for three main conclusions. Firstly, this work shows that there are a range of expressions that communicate positive emotions. The study of a wide range of nonverbal cues is crucial, as many emotions are only reliably communicated via one channel of communication or a combination of channels. Secondly, the reviewed results demonstrate that there are a number of positive emotions that are associated with unique, recognisable signals. Specifically, the fact that many of the studies reviewed have found evidence of recognition of positive emotions, when examining them in the context of other positive emotions, attests to the signal clarity of these expressions (see Table 2). Thirdly, the available findings indicate that the notion of emotion families may be useful as a way to distinguish between positive emotions, based on the associated nonverbal behaviour (see Ellsworth & Smith, 1988, for evidence based on appraisal profiles). The epistemological positive emotions (amusement, relief, awe, and interest) have distinct, recognisable displays via vocal or facial cues. The recognisability of these signals suggests that the nonverbal communication of these emotional states may be adaptive in an evolutionary sense. They may, together with pride, be the most likely candidates for potentially basic positive emotions, equivalent to the set of negative emotions that are reliably communicated via nonverbal signals (e.g., fear, disgust, anger). The agency-approach positive emotions (elation and pride) appear to be associated with recognisable visual, but not auditory, cues. This could indicate that these emotions are primarily communicated with others in relatively close proximity, since auditory cues (e.g., screams) may travel further than visual ones (e.g., eyebrow raising). However, the relatively conspicuous bodily cues associated with both elation and pride are indicative of signals that could be inferred from others from a distance. Furthermore, there is limited data on the production of vocal cues associated with elation and pride; hopefully future studies will explore this issue. Evidence is less strong for clear signals of the prosocial emotions (love, compassion, gratitude, and admiration) in any modality other than touch. This is consistent with previous findings that have highlighted the preferential communication of prosocial emotions via touch and is thought to reflect the fact that these emotions primarily occur in intimate relationships (App et al., 2011). Finally, there is little support for distinct recognisable signals of the savouring positive emotions (contentment, sensory pleasure, and desire), which may suggest that these emotions are not associated with communicative functions, though they may nevertheless serve adaptive functions for the individual who is experiencing them.

The suggestion of some consistencies in patterns of results within the proposed emotion families is not intended to imply that these categories are necessarily the best possible conceptualisation of positive emotion space. Nevertheless, current evidence points to possible superordinate classes of positive emotions, in addition to underlining the differentiation between specific positive emotional states. It is also worth noting that a number of positive emotions have not been studied at all yet in the context of nonverbal behaviour; future work may for example examine the communication of ecstasy, devotion, and hope.

These findings overturn some previous assumptions about the nonverbal communication of positive emotions. Specifi-cally, all positive emotions were once thought to share a nonverbal signal in the form of a smile (e.g., Ekman, 1992). It is well established that smiling does not always provide a direct read-out of felt enjoyment (e.g., Kraut & Johnston, 1979), but the findings reviewed here go beyond this point to reveal that some positive emotions, like interest, are reliably expressed and recognised by facial cues other than smiling. In addition, some positive emotional states are reliably communicated via nonfacial signals. Smiling may nevertheless provide a signal of general positive emotion or prosocial orientation, but the available data suggests that more detailed characterisations of configurations of cues are likely more informative than using single, global descriptive categories such as smiling.

The present review included only studies with adult participants, but it is worth considering how this work connects to the developmental literature (reviewed in Sauter et al., 2014). It is well established that already very early in development, infants are sensitive to nonverbal signals of positive emotion from others and also that they produce such signals themselves. There is, for example, evidence that infants produce laughter in response to playful physical games. Notably, similarities between developmental and adult samples have been found across multiple modalities. Even very young infants show differentiation between different kinds of smiles in both perception and production, but little work focusing on early development has included comparisons between multiple kinds of positive emotions. Research on emotion perception in older children has in some cases included several positive emotions and shown considerable differentiation, but data on many aspects of the development of nonverbal communication of specific positive emotions are lacking. Addressing this gap in the research literature will be a worthwhile challenge for future research.

It is worth noting some limitations of the extant data, particularly as this may inform further research pursuits in this field. There is a great need for more cross-cultural research in the study of nonverbal communication of positive emotions. With the exception of a handful of studies noted in the relevant sections, the displays of most positive emotions have not been tested across cultures for any channel of communication. It is well established that cultures vary in their orientations to different types of positive affect (Tsai, Knutson, & Fung, 2006), but the extent to which culture shapes emotional signals varies greatly across positive emotions (e.g., Sauter, Eisner, Ekman, et al., 2010). More research in this area is thus needed, particularly given the importance of cross-cultural findings for evolutionary arguments on emotions.

A number of studies have demonstrated a communication advantage for multimodal expressions (e.g., Bänziger et al., 2012). The vast majority of studies to date have focused on expressions within a single modality. Research on unimodal communication can provide a tough test of emotion recognition, but including multimodal expressions will be an important next step to increasing ecological validity. Relatedly, it is an obvious limitation that so much of this research relies on posed emotional expressions; here the study of positive emotions has an advantage compared to that of negative emotions in that there are generally fewer ethical concerns with inducing intense emotional experiences in the laboratory.

Finally, there is enormous variability in terms of the measures, target emotions, and paradigms in the studies conducted to date. Although open exploration is crucial for discovery, hopefully the maturation of this field of research will begin to yield greater convergence on methods and emotions over the coming years.
